# Genomic Responses to Climate Change: Making the Most of the *Drosophila* Model

**DOI:** 10.3389/fgene.2021.676218

**Published:** 2021-07-13

**Authors:** Murillo F. Rodrigues, Rodrigo Cogni

**Affiliations:** ^1^Institute of Ecology and Evolution, University of Oregon, Eugene, OR, United States; ^2^Department of Ecology, Institute of Biosciences, University of São Paulo, São Paulo, Brazil

**Keywords:** cline, wild populations, global warming, genomic adaptation, climate adaptation, natural selection, *Drosophila*, historical samples

## Abstract

It is pressing to understand how animal populations evolve in response to climate change. We argue that new sequencing technologies and the use of historical samples are opening unprecedented opportunities to investigate genome-wide responses to changing environments. However, there are important challenges in interpreting the emerging findings. First, it is essential to differentiate genetic adaptation from phenotypic plasticity. Second, it is extremely difficult to map genotype, phenotype, and fitness. Third, neutral demographic processes and natural selection affect genetic variation in similar ways. We argue that *Drosophila melanogaster*, a classical model organism with decades of climate adaptation research, is uniquely suited to overcome most of these challenges. In the near future, long-term time series genome-wide datasets of *D. melanogaster* natural populations will provide exciting opportunities to study adaptation to recent climate change and will lay the groundwork for related research in non-model systems.

## Introduction

Variation in environmental conditions across space can result in local adaptation, where populations are more fit to the environment in which they evolved (Kawecki and Ebert, 2004). With climatic conditions changing at an alarming rate (Diffenbaugh et al., 2018), populations will need to migrate to more favorable climates, modify their phenotypes via plasticity or evolutionary change, or face extinction (Hoffmann and Sgro, 2011; Waldvogel et al., 2020). Thus, understanding the causes and consequences of local climate adaptation is fundamental to predict species responses to future changes in climate.

A promising way to understand climate adaptation lies in the use of historical samples. Given the historical nature of the evolutionary process, samples from multiple timepoints can help better identify the targets of selection. Combined with recent advances in sequencing technologies, it is now possible to compare entire genomes of present and past populations (Leonardi et al., 2016). For example, Alves et al. (2019) used modern and historical rabbit specimens to identify the genetic basis of resistance to myxoma virus, which decimated rabbit populations in the 1950s. Likewise, Bi et al. (2019) sequenced modern and historic museum specimens to investigate the role of climate change in driving genomic changes over the last century in two chipmunk species.

Historical samples can also inform long-standing debates in evolutionary biology, such as whether adaptation is limited by the supply of mutations (Karasov et al., 2010) or whether migration can facilitate adaptation (Pennings and Hermisson, 2006). With historical samples, it would be possible to watch the sweep of beneficial mutations in action. If adaptation is limited by mutations, then we would expect swept alleles to be rare before they sweep through the population (Feder et al., 2021). If multiple populations are sampled, then you could ask whether alleles that sweep through one population were already present in other populations. Both of these questions are essential to understanding the impact that climate change will have on species, and can be useful for designing conservation strategies (Matz et al., 2018) or predicting species’ responses to climate change (Fuller et al., 2020).

Although these advances present exciting opportunities to understand local climate adaptation and better predict responses to climate change, there are important challenges in obtaining and interpreting historical genomic datasets. First, it is critical to discern between genetic adaptation and phenotypic plasticity. Second, it remains difficult to map genotype, phenotype, and fitness. Third, it is hard to disentangle the role of adaptive and demographic processes in shaping genetic variation. In this perspective article, we discuss these challenges and argue that classical model organisms, such as *Drosophila melanogaster*, are uniquely suited to overcome most of these difficulties.

## *Drosophila melanogaster* and Climate Adaptation

**FIGURE 1:**
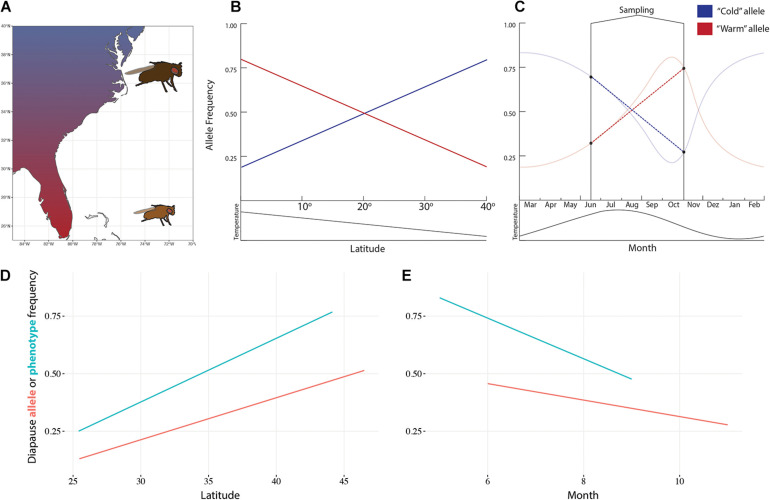
Spatial and temporal variation in *Drosophila melanogaster*. **(A)** Flies vary in size, color, and many other phenotypic and genotypic traits across the east coast of the United States. Alleles related to climate adaptation are expected to be correlated with latitude **(B)** and seasons **(C)**. For example, diapause incidence and the frequency of an allele that encodes for diapause inducibility in the *couchpotato* gene vary predictably with latitude **(D)** and seasons **(E)**. Data from Schmidt et al. (2005) and Cogni et al. (2014).

*Drosophila melanogaster* is native to southern-central Africa and has adapted to temperate climates around the world. It colonized Europe around 1,400 years ago, and North America and Australia around 150 years ago (Keller, 2007; Sprengelmeyer et al., 2020). *D. melanogaster* climate adaptation has been intensely studied over the last few decades. Natural fly populations show clear latitudinal patterns at the phenotypic and genotypic level (Figure 1). Flies from higher latitudes are bigger, darker, more stress tolerant, and show higher incidence of reproductive diapause than populations from lower latitudes (Adrion et al., 2015). Similar patterns of variation have been observed at seasonal and altitudinal scales (e.g., Pitchers et al., 2013; Bergland et al., 2014; Behrman et al., 2018).

*Drosophila* is an amenable model to study genetics of climate adaptation not only because of its peculiar colonization history. Species in this group have short generation times and can be easily reared in the laboratory. This allows for experimental validation; for instance, one can measure fitness of flies from different populations under various conditions to directly test selection hypotheses (Fabian et al., 2015). More recently, studies have shown that it is even possible to rear flies in their natural environment, with outdoor cages (Rudman et al., 2019). *Drosophila* is a remarkably diverse group, with almost unparalleled genomic resources (e.g., Kim et al., 2020), and so comparative genomics studies are within reach. For instance, by comparing *D. melanogaster* and *Drosophila simulans*, Machado et al. (2016) found that the ability to overwinter is necessary for adapting to temperate environments.

Spatiotemporal sampling efforts are unparalleled in *D. melanogaster*. Because flies have short generation times, the system is highly compatible with empirical time series studies. Time series studies can differ in depth of past evolutionary history. Dobzhansky (1943) demonstrated that some inversions cycle with the seasons in *Drosophila pseudoobscura*. In *D. melanogaster*, many variants seem to cycle seasonally in a single temperate population (Bergland et al., 2014) as well as across many different locations worldwide (Machado et al., 2021). Two consortiums in Europe and North America, DrosEU and the DrosRTEC, have been sampling and sequencing wild *D. melanogaster* populations annually since 2014 across multiple locations (Kapun et al., 2020, 2021; Machado et al., 2021). These efforts are expected to continue for the foreseeable future, increasing even further spatiotemporal resolution. Other studies went even far back in time and compared classical latitudinal patterns published decades ago with more recent data (Umina et al., 2005; Cogni et al., 2014, 2017). Efforts to sequence specimens collected decades ago are just now starting to emerge (Veeramah et al., 2020; John Pool, personal communication). Theoretically, one could go even deeper in time by sequencing museum specimens (Wandeler et al., 2007), but it is unclear whether museums have enough disposable specimens. Future advances in DNA sequencing with minimal tissue availability could make this more feasible.

## Disentangling Genetic Adaptation and Phenotypic Plasticity

Phenotypic plasticity can challenge interpretation of climate adaptation. Most of the examples of biological responses to climate change observed for a wide range of organisms seem to be due to phenotypic plasticity and not adaptation to a new environment (Parmesan, 2006; Gienapp et al., 2008; Merilä and Hendry, 2014). In a recent study, both plasticity and genetic changes were found to contribute to climate change response (Ramakers et al., 2019). Thus, to understand how adaptation can contribute to organisms’ resilience to climate change, it is crucial to discern between plasticity and genetic adaptation as the cause of trait changes (Kellermann and van Heerwaarden, 2019; Kellermann et al., 2020).

In *Drosophila*, plasticity and adaptation can be distinguished with two different approaches. First, lineages from divergent populations can be reared in the laboratory under similar environmental conditions. Plastic phenotype responses are expected to disappear when divergent populations are reared in the same conditions, whereas genetic adaptations should persist (Ayrinhac et al., 2004; Mitchell et al., 2011). Selection experiments in the laboratory can be used to quantify the contributions of phenotypic plasticity and genetic adaptation (Garland and Kelly, 2006). With these experiments, it is possible to exert selection on a single environmental condition (e.g., temperature) and measure responses at the phenotypic and genomic levels (Barghi et al., 2019). Then, the adapted lineages can be reared under different conditions to tease apart the role of phenotypic plasticity. Climate adaptation can be complex, making it difficult to be addressed in laboratory settings which are known to have many subtle environmental differences. However, recent *Drosophila* studies have shown that flies can be raised in outdoor cages, under conditions which are remarkably similar to those which natural wild populations experience (Rudman et al., 2019, 2021).

Second, genetic adaptation can be disentangled from phenotypic plasticity by studying changes at the molecular level, that is with direct observation of allele frequency changes in natural populations. For example, Umina et al. (2005) observed a shift over 20 years in a classic *D. melanogaster* latitudinal cline in the alcohol dehydrogenase polymorphism. The frequency of the warmer-adapted allele increased in frequency along the cline, presumably in response to warmer and drier conditions. There are also examples in other *Drosophila* species of changes in chromosome inversion frequencies correlated with climate warming (Balanya et al., 2006, 2009; Batista et al., 2012). These direct observations of genetic changes can refute the phenotypic plasticity hypotheses. However, it is not clear if the observed shifts reflected changes in local selection, a progressive invasion of warm-adapted genotypes from lower latitudes/altitudes, or a combination of both (Balanya et al., 2006).

## Connecting Genomic Variation to Phenotypes and Fitness

Another major challenge is to connect segregating genomic variation to variation in phenotypes and fitness. Even in the best studied model species, examples of phenotypes that were mapped to major effect loci are scarce (e.g., Schmidt et al., 2008) (but see Erickson et al., 2020). For most clinal phenotypes in *D. melanogaster*, such as body size and longevity, the underlying genetic architecture is more complex, with many minor effect alleles explaining only a small proportion of the phenotypic variation (Mackay et al., 2012). One would expect that the variants that underlie clinal traits might display clinal patterns in their allele frequencies. This approach has been successfully used in humans where height-increasing alleles are systematically elevated in frequency in the north compared to southern European populations (Berg and Coop, 2014), and in *D. melanogaster* where polymorphisms associated with diapause varies clinally (Erickson et al., 2020). However, in another *D. melanogaster* example, the frequency of polymorphisms associated with desiccation tolerance does not vary clinally as expected (Rajpurohit et al., 2018). Other phenotypes have not yet been tested, but if results similar to the desiccation example are observed, it could indicate low statistical power on genome-wide association studies or complex epistatic and genotype-by-environment interactions.

In *D. melanogaster*, these challenges can be overcome in a few ways. New panels with much larger number of recombinant inbred lines can substantially increase the power of GWAS studies to identify minor effect alleles and epistatic interactions (King et al., 2012; Cogni et al., 2016). Further, there exist diverse collections of wild-caught flies, which can be used in mapping and experimental evolution studies (Grenier et al., 2015; Lack et al., 2016). By harnessing the immense natural variation in *D. melanogaster*, mapping power can be greatly increased (Gasch et al., 2016). The system also allows for laboratory experiments under different environmental conditions, and genotype-by-environment interactions can be directly measured (e.g., Lazzaro et al., 2008).

**FIGURE 2:**
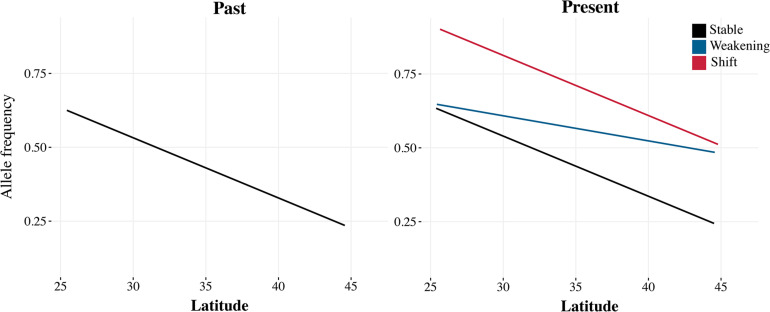
In organisms with well-characterized patterns of clinal genomic variation, we can make clear predictions on the expected shifts in allele frequencies due to climate change. With strong climate change, clines can weaken (blue) so that the frequency of a warm-adapted allele increases in higher latitudes; or shift (red) so that the frequency of the warm-adapted allele increases along the cline. If the change in climate is not strong, but the cline is being maintained by selection, clines should be more stable (black).

An alternative to the major challenges in genotype-to-phenotype mapping would be to rely on already known instances of climate adaptation in *D. melanogaster*. Genome-wide patterns of clinal, altitudinal, and seasonal adaptation have been well characterized in the system, so we can make clear predictions on the shifts in allele frequencies expected by climate change, even without linking genotypes to particular phenotypes. Alleles with higher frequency in warm regions are expected to increase in frequency as the climate becomes warmer over time (Figure 2; Umina et al., 2005; Cogni et al., 2017).

## Differentiating Neutral and Adaptive Processes

The final challenge is that inferring the role of selection in shaping genetic variation in natural populations is not trivial. If selection is pushing traits to different optima across space, the loci underlying the selected traits should be excessively differentiated between populations (Endler, 1977). Similarly, environmental conditions that determine fitness are expected to be correlated with allele frequency at the relevant loci (Figure 1) (Kawecki and Ebert, 2004). Although these features of genomic data are indicative of local adaptation, the evidence is correlative in nature and may be confounded by non-adaptive processes. For example, demographic processes alone can create correlations between allele frequencies and the environment (Caracristi and Schlötterer, 2003).

One way to strengthen the hypothesis of adaptation is to look for parallel patterns of variation in different continents, between altitude and latitude, and between latitude and season (Paaby et al., 2010; Klepsatel et al., 2014; Cogni et al., 2015; Rodrigues et al., 2020). However, much of adaptation may not result in parallelism due to redundancy (Barghi et al., 2019). That is, particularly for polygenic traits there may be many alternative ways of reaching an adaptive peak. For example, couch potato clines associated with diapause are observed in North America, but not in Australia (Lee et al., 2011; Cogni et al., 2014). *D. melanogaster* is nonetheless a great system to dissect the genetic bases of adaptation in detail (Tauber et al., 2007; Schmidt et al., 2008; Erickson et al., 2020).

Other features of genomic data can be used to disentangle demography and selection. Most importantly, selection alters not only allele frequencies but also haplotype frequencies. Indeed, it is possible to discern between different demographic scenarios and models of selection with haplotype data (Garud et al., 2021). One can obtain phased genomic data more easily for *Drosophila*, either by sequencing inbred lines (which are mostly homozygous) or haploid embryos (Langley et al., 2011).

Historical sampling can help differentiate the role of adaptive and non-adaptive forces in driving changes over time and across space. With historical samples, it is possible to directly measure the rate of allele frequency change, which is expected to be particularly high for loci under selection. A few methods have been developed to use time series allele frequency data to detect loci evolving non-neutrally (reviewed in Malaspinas, 2016), but more powerful methods that consider other features of genomic data, such as haplotypes, have yet to be developed.

The stability of trait–environment correlations, which is a hallmark of local adaptation, can also be assessed with historical samples (Figure 2). In the short term, stable correlations are strong evidence for natural selection (Cogni et al., 2014), because spurious correlations due to demography are expected to be transient. However, as populations respond to changing climate conditions, we expect the underlying trait–environment correlations to change as well. Depending on how climate change affects fitness, clines can become less steep, with flies from high latitudes becoming more similar to flies from low latitudes or shift entirely so that the frequency of the warm adapted allele increases across the entire range. In the case of *D. melanogaster*, it would be interesting to quantify the effect of climate change on local adaptation by looking specifically at loci that are known to be relevant to climate adaptation, such as the inversion 3R Payne or the loci underlying diapause and metabolism (Cogni et al., 2014; Lavington et al., 2014; Siddiq and Thornton, 2019). Have clines at these loci weakened or shifted in the past 50 years?

## Conclusion

Time series genomic datasets are bound to help the study of climate adaptation. Indeed, historical samples have been recently used to study global environmental change (Key et al., 2016; Lang et al., 2019). Here, we discussed some of the major challenges in studying adaptation with modern and historical genomic data. We argued that the decades of climate adaptation research make *Drosophila* an ideal system to study genomic responses to climate change using historical samples. In the coming years, the emergence of temporal genomic datasets will provide exciting opportunities to study adaptation to climate in *Drosophila* (Kapun et al., 2020; Veeramah et al., 2020; Machado et al., 2021) and will lay the groundwork for similar approaches in non-model systems.

## Data Availability Statement

The original contributions presented in the study are included in the article/supplementary material, further inquiries can be directed to the corresponding author/s.

## Author Contributions

MR and RC wrote the manuscript. Both authors contributed to the article and approved the submitted version.

## Conflict of Interest

The authors declare that the research was conducted in the absence of any commercial or financial relationships that could be construed as a potential conflict of interest.

## References

[B1] Adrion J. R. Hahn M. W. Cooper B. S. (2015). Revisiting classic clines in *Drosophila melanogaster* in the age of genomics. *Trends Genet.* 31 434–444. 10.1016/j.tig.2015.05.006 26072452 PMC4526433

[B2] Alves J. M. Carneiro M. Cheng J. Y. De Matos A. L. Rahman M. M. Loog L. (2019). Parallel adaptation of rabbit populations to myxoma virus. *Science* 363 1319–1326. 30765607 10.1126/science.aau7285 PMC6433279

[B3] Ayrinhac A. Debat V. Gibert P. Kister A.-G. Legout H. Moreteau B. (2004). Cold adaptation in geographical populations of *Drosophila melanogaster*: phenotypic plasticity is more important than genetic variability. *Funct. Ecol.* 18 700–706. 10.1111/j.0269-8463.2004.00904.x

[B4] Balanya J. Huey R. B. Gilchrist G. W. Serra L. (2009). The chromosomal polymorphism of *Drosophila subobscura*: a microevolutionary weapon to monitor global change. *Heredity* 103 364–367. 10.1038/hdy.2009.86 19639003

[B5] Balanya J. Oller J. M. Huey R. B. Gilchrist G. W. Serra L. (2006). Global genetic change tracks global climate warming in *Drosophila subobscura*. *Science* 313 1773–1775. 10.1126/science.1131002 16946033

[B6] Barghi N. Tobler R. Nolte V. Jakšić A. M. Mallard F. Otte K. A. (2019). Genetic redundancy fuels polygenic adaptation in *Drosophila*. *PLoS Biol.* 17:e3000128. 10.1371/journal.pbio.3000128 30716062 PMC6375663

[B7] Batista M. R. D. Ananina G. Klaczko L. B. (2012). Unexpected long-term changes in chromosome inversion frequencies in a Neotropical *Drosophila* species. *Clim. Res.* 53 131–140. 10.3354/cr01088

[B8] Behrman E. L. Howick V. M. Kapun M. Staubach F. Bergland A. O. Petrov D. A. (2018). Rapid seasonal evolution in innate immunity of wild *Drosophila melanogaster*. *Proc. Biol. Sci.* 285:20172599. 10.1098/rspb.2017.2599 29321302 PMC5784205

[B9] Berg J. J. Coop G. (2014). A Population Genetic Signal of Polygenic Adaptation. *PLoS Genet.* 10:e1004412. 10.1371/journal.pgen.1004412 25102153 PMC4125079

[B10] Bergland A. O. Behrman E. L. O’brien K. R. Schmidt P. S. Petrov D. A. (2014). Genomic evidence of rapid and stable adaptive oscillations over seasonal time scales in *Drosophila*. *PLoS Genet.* 10:e1004775. 10.1371/journal.pgen.1004775 25375361 PMC4222749

[B11] Bi K. Linderoth T. Singhal S. Vanderpool D. Patton J. L. Nielsen R. (2019). Temporal genomic contrasts reveal rapid evolutionary responses in an alpine mammal during recent climate change. *PLoS Genet.* 15:e1008119. 10.1371/journal.pgen.1008119 31050681 PMC6519841

[B12] Caracristi G. Schlötterer C. (2003). Genetic Differentiation Between American and European *Drosophila melanogaster* Populations Could Be Attributed to Admixture of African Alleles. *Mol. Biol. Evol.* 20 792–799. 10.1093/molbev/msg091 12679536

[B13] Cogni R. Cao C. Day J. P. Bridson C. Jiggins F. M. (2016). The genetic architecture of resistance to virus infection in *Drosophila*. *Mol. Ecol.* 25 5228–5241. 10.1111/mec.13769 27460507 PMC5082504

[B14] Cogni R. Kuczynski C. Koury S. Lavington E. Behrman E. L. O’brien K. R. (2014). The Intensity of Selection Acting on the Couch Potato Gene-Spatial-Temporal Variation in a Diapause Cline. *Evolution* 68 538–548. 10.1111/evo.12291 24303812

[B15] Cogni R. Kuczynski K. Koury S. Lavington E. Behrman E. L. O’brien K. R. (2017). On the Long-term Stability of Clines in Some Metabolic Genes in *Drosophila melanogaster*. *Sci. Rep.* 7:42766. 10.1038/srep42766 PMC5318857 28220806

[B16] Cogni R. Kuczynski K. Lavington E. Koury S. Behrman E. L. O’brien K. R. (2015). Variation in *Drosophila melanogaster* central metabolic genes appears driven by natural selection both within and between populations. *Proc. Biol. Sci.* 282:20142688. 10.1098/rspb.2014.2688 25520361 PMC4298213

[B17] Diffenbaugh N. S. Singh D. Mankin J. S. (2018). Unprecedented climate events: historical changes, aspirational targets, and national commitments. *Sci. Adv.* 4:eaao3354. 10.1126/sciadv.aao3354 29457133 PMC5812734

[B18] Dobzhansky T. (1943). Genetics of Natural Populations IX. Temporal Changes in the Composition of Populations of *Drosophila Pseudoobscura*. *Genetics* 28 162–186. 10.1093/genetics/28.2.162 17247077 PMC1209199

[B19] Endler J. A. (1977). *Geographic Variation, Speciation, and Clines.* New Jersey: Princeton University Press. 409931

[B20] Erickson P. A. Weller C. A. Song D. Y. Bangerter A. S. Schmidt P. Bergland A. O. (2020). Unique genetic signatures of local adaptation over space and time for diapause, an ecologically relevant complex trait, in *Drosophila melanogaster*. *PLoS Genet.* 16:e1009110. 10.1371/journal.pgen.1009110 33216740 PMC7717581

[B21] Fabian D. K. Lack J. B. Mathur V. Schlotterer C. Schmidt P. S. Pool J. E. (2015). Spatially varying selection shapes life history clines among populations of *Drosophila melanogaster* from sub-Saharan Africa. *J. Evol. Biol.* 28 826–840. 10.1111/jeb.12607 25704153 PMC4405473

[B22] Feder A. F. Pennings P. S. Petrov D. A. (2021). The clarifying role of time series data in the population genetics of HIV. *PLoS Genet.* 17:e1009050. 10.1371/journal.pgen.1009050 33444376 PMC7808693

[B23] Fuller Z. L. Mocellin V. J. L. Morris L. A. Cantin N. Shepherd J. Sarre L. (2020). Population genetics of the coral *Acropora millepora*: toward genomic prediction of bleaching. *Science* 369:eaba4674. 10.1126/science.aba4674 32675347

[B24] Garland T. Jr. Kelly S. A. (2006). Phenotypic plasticity and experimental evolution. *J. Exp. Biol.* 209 2344–2361. 16731811 10.1242/jeb.02244

[B25] Garud N. R. Messer P. W. Petrov D. A. (2021). Detection of hard and soft selective sweeps from *Drosophila melanogaster* population genomic data. *PLoS Genet.* 17:e1009373. 10.1371/journal.pgen.1009373 33635910 PMC7946363

[B26] Gasch A. P. Payseur B. A. Pool J. E. (2016). The Power of Natural Variation for Model Organism Biology. *Trends Genet.* 32 147–154. 10.1016/j.tig.2015.12.003 26777596 PMC4769656

[B27] Gienapp P. Teplitsky C. Alho J. S. Mills J. A. Merilä J. (2008). Climate change and evolution: disentangling environmental and genetic responses. *Mol. Ecol.* 17 167–178. 10.1111/j.1365-294x.2007.03413.x 18173499

[B28] Grenier J. K. Arguello J. R. Moreira M. C. Gottipati S. Mohammed J. Hackett S. R. (2015). Global diversity lines - a five-continent reference panel of sequenced *Drosophila melanogaster* strains. *G3 (Bethesda)* 5 593–603. 10.1534/g3.114.015883 25673134 PMC4390575

[B29] Hoffmann A. A. Sgro C. M. (2011). Climate change and evolutionary adaptation. *Nature* 470 479–485. 21350480 10.1038/nature09670

[B30] Kapun M. Barrón M. G. Staubach F. Obbard D. J. Wiberg R. A. W. Vieira J. (2020). Genomic Analysis of European *Drosophila melanogaster* Populations Reveals Longitudinal Structure, Continent-Wide Selection, and Previously Unknown DNA Viruses. *Mol. Biol. Evol.* 37 2661–2678. 10.1093/molbev/msaa120 32413142 PMC7475034

[B31] Kapun M. Nunez J. C. B. Bogaerts-Márquez M. Murga-Moreno J. Paris M. Outten J. (2021). *Drosophila* Evolution over Space and Time (DEST) - A New Population Genomics Resource. *bioRxiv* [Preprint]. 10.1101/2021.02.01.428994 PMC8662648 34469576

[B32] Karasov T. Messer P. W. Petrov D. A. (2010). Evidence that adaptation in *Drosophila* is not limited by mutation at single sites. *PLoS Genet.* 6:e1000924. 10.1371/journal.pgen.1000924 20585551 PMC2887467

[B33] Kawecki T. J. Ebert D. (2004). Conceptual issues in local adaptation. *Ecol. Lett.* 7 1225–1241. 10.1111/j.1461-0248.2004.00684.x

[B34] Keller A. (2007). *Drosophila melanogaster*’s history as a human commensal. *Curr. Biol.* 17 R77–81. 17276902 10.1016/j.cub.2006.12.031

[B35] Kellermann V. Mcevey S. F. Sgrò C. M. Hoffmann A. A. (2020). Phenotypic plasticity for desiccation resistance, climate change, and future species distributions: will plasticity have much impact? *Am. Nat.* 196 306–315. 10.1086/710006 32814000

[B36] Kellermann V. van Heerwaarden B. (2019). Terrestrial insects and climate change: adaptive responses in key traits. *Physiol. Entomol.* 44 99–115. 10.1111/phen.12282

[B37] Key F. M. Fu Q. Romagné F. Lachmann M. Andrés A. M. (2016). Human adaptation and population differentiation in the light of ancient genomes. *Nat. Commun.* 7:10775. 10.1038/ncomms10775 PMC4802047 26988143

[B38] Kim B. Y. Wang J. R. Miller D. E. Barmina O. Delaney E. Thompson A. (2020). Highly contiguous assemblies of 101 drosophilid genomes. *bioRxiv* [Preprint]. 10.1101/2020.12.14.422775 PMC8337076 34279216

[B39] King E. G. Macdonald S. J. Long A. D. (2012). Properties and power of the *Drosophila* Synthetic Population Resource for the routine dissection of complex traits. *Genetics* 191 935–949. 10.1534/genetics.112.138537 22505626 PMC3389985

[B40] Klepsatel P. Gáliková M. Huber C. D. Flatt T. (2014). Similarities and differences in altitudinal versus latitudinal variation for morphological traits in *Drosophila melanogaster*. *Evolution* 68 1385–1398. 10.1111/evo.12351 24410363

[B41] Langley C. H. Crepeau M. Cardeno C. Corbett-Detig R. Stevens K. (2011). Circumventing heterozygosity: sequencing the amplified genome of a single haploid Drosophila melanogaster embryo. *Genetics*, 188 239–246. 10.1534/genetics.111.127530 21441209 PMC3122310

[B42] Lack J. B. Lange J. D. Tang A. D. Corbett-Detig R. B. Pool J. E. (2016). A Thousand Fly Genomes: an expanded *drosophila* genome nexus. *Mol. Biol. Evol.* 33 3308–3313. 10.1093/molbev/msw195 27687565 PMC5100052

[B43] Lang P. L. M. Willems F. M. Scheepens J. F. Burbano H. A. Bossdorf O. (2019). Using herbaria to study global environmental change. *New Phytol.* 221 110–122. 10.1111/nph.15401 30160314 PMC6585664

[B44] Lavington E. Cogni R. Kuczynski C. Koury S. Behrman E. L. O’brien K. R. (2014). A small system–high-resolution study of metabolic adaptation in the central metabolic pathway to temperate climates in *Drosophila melanogaster*. *Mol. Biol. Evol.* 31 2032–2041. 10.1093/molbev/msu146 24770333 PMC4104311

[B45] Lazzaro B. P. Flores H. A. Lorigan J. G. Yourth C. P. (2008). Genotype-by-environment interactions and adaptation to local temperature affect immunity and fecundity in *Drosophila melanogaster*. *PLoS Pathog.* 4:e1000025. 10.1371/journal.ppat.1000025 18369474 PMC2265416

[B46] Lee S. F. Sgrò C. M. Shirriffs J. Wee C. W. Rako L. Van Heerwaarden B. (2011). Polymorphism in the couch potato gene clines in eastern Australia but is not associated with ovarian dormancy in *Drosophila melanogaster*. *Mol. Ecol.* 20 2973–2984. 10.1111/j.1365-294x.2011.05155.x 21689187

[B47] Leonardi M. Librado P. Der Sarkissian C. Schubert M. Alfarhan A. H. Alquraishi S. A. (2016). Evolutionary patterns and processes: lessons from Ancient DNA. *Syst. Biol.* 66 e1–29. 10.1093/sysbio/syw059 28173586 PMC5410953

[B48] Machado H. E. Bergland A. O. O’brien K. R. Behrman E. L. Schmidt P. S. Petrov D. A. (2016). Comparative population genomics of latitudinal variation in *Drosophila* simulans and *Drosophila melanogaster*. *Mol. Ecol.* 25 723–740. 10.1111/mec.13446 26523848 PMC5089931

[B49] Machado H. E. Bergland A. O. Taylor R. Tilk S. Behrman E. Dyer K. (2021). Broad geographic sampling reveals predictable, pervasive, and strong seasonal adaptation in *Drosophila*. *bioRxiv* [Preprint]. 10.1101/337543 PMC8248982 34155971

[B50] Mackay T. F. C. Richards S. Stone E. A. Barbadilla A. Ayroles J. F. Zhu D. H. (2012). The *Drosophila melanogaster* Genetic Reference Panel. *Nature* 482 173–178. 22318601 10.1038/nature10811 PMC3683990

[B51] Malaspinas A. S. (2016). Methods to characterize selective sweeps using time serial samples: an ancient DNA perspective. *Mol. Ecol.* 25 24–41. 10.1111/mec.13492 26613371

[B52] Matz M. V. Treml E. A. Aglyamova G. V. Bay L. K. (2018). Potential and limits for rapid genetic adaptation to warming in a Great Barrier Reef coral. *PLoS Genet.* 14:e1007220. 10.1371/journal.pgen.1007220 29672529 PMC5908067

[B53] Merilä J. Hendry A. P. (2014). Climate change, adaptation, and phenotypic plasticity: the problem and the evidence. *Evol. Appl.* 7 1–14. 10.1111/eva.12137 24454544 PMC3894893

[B54] Mitchell K. A. Sgrò C. M. Hoffmann A. A. (2011). Phenotypic plasticity in upper thermal limits is weakly related to *Drosophila* species distributions. *Funct. Ecol.* 25 661–670. 10.1111/j.1365-2435.2010.01821.x

[B55] Paaby A. B. Blacket M. J. Hoffmann A. A. Schmidt P. S. (2010). Identification of a candidate adaptive polymorphism for *Drosophila* life history by parallel independent clines on two continents. *Mol. Ecol.* 19 760–774. 10.1111/j.1365-294x.2009.04508.x 20074316

[B56] Parmesan C. (2006). Ecological and evolutionary responses to recent climate change. *Annu. Rev. Ecol. Evol. Syst.* 37 637–669. 10.1146/annurev.ecolsys.37.091305.110100

[B57] Pennings P. S. Hermisson J. (2006). Soft sweeps II–molecular population genetics of adaptation from recurrent mutation or migration. *Mol. Biol. Evol.* 23 1076–1084. 10.1093/molbev/msj117 16520336

[B58] Pitchers W. Pool J. E. Dworkin I. (2013). Altitudinal clinal variation in wing size and shape in african *Drosophila melanogaster* : one cline or many? *Evolution* 67 438–452. 10.1111/j.1558-5646.2012.01774.x 23356616 PMC3786396

[B59] Rajpurohit S. Gefen E. Bergland A. O. Petrov D. A. Gibbs A. G. Schmidt P. S. (2018). Spatiotemporal dynamics and genome-wide association genome-wide association analysis of desiccation tolerance in *Drosophila melanogaster*. *Mol. Ecol.* 27 3525–3540. 10.1111/mec.14814 30051644 PMC6129450

[B60] Ramakers J. J. C. Gienapp P. Visser M. E. (2019). Phenological mismatch drives selection on elevation, but not on slope, of breeding time plasticity in a wild songbird. *Evolution* 73 175–187. 10.1111/evo.13660 30556587 PMC6519030

[B61] Rodrigues M. F. Vibranovski M. D. Cogni R. (2020). Natural selection and parallel clinal and seasonal changes in *Drosophila melanogaster*. *bioRxiv* [Preprint]. 10.1101/2020.03.19.999011 34184262

[B62] Rudman S. M. Greenblum S. Hughes R. C. Rajpurohit S. Kiratli O. Lowder D. B. (2019). Microbiome composition shapes rapid genomic adaptation of *Drosophila melanogaster*. *Proc. Natl. Acad. Sci. U. S. A.* 116 20025–20032. 10.1073/pnas.1907787116 31527278 PMC6778213

[B63] Rudman S. M. Greenblum S. I. Rajpurohit S. Betancourt N. J. Hanna J. Tilk S. (2021). Direct observation of adaptive tracking on ecological timescales in *Drosophila*. *bioRxiv* [Preprint]. 10.1101/2021.04.27.441526 PMC10684103 35298245

[B64] Schmidt P. S. Matzkin L. Ippolito M. Eanes W. F. (2005). Geographic variation in diapause incidence, life-history traits, and climatic adaptation in *Drosophila melanogaster*. *Evolution* 59 1721–1732. 10.1111/j.0014-3820.2005.tb01821.x 16331839

[B65] Schmidt P. S. Zhu C. T. Das J. Batavia M. Yang L. Eanes W. F. (2008). An amino acid polymorphism in the couch potato gene forms the basis for climatic adaptation in *Drosophila melanogaster*. *Proc. Natl. Acad. Sci. U. S. A.* 105 16207–16211. 10.1073/pnas.0805485105 18852464 PMC2570987

[B66] Siddiq M. A. Thornton J. W. (2019). Fitness effects but no temperature-mediated balancing selection at the polymorphic *Adh* gene of *Drosophila melanogaster*. *Proc. Natl. Acad. Sci.* 116 21634–21640. 31594844 10.1073/pnas.1909216116 PMC6815130

[B67] Sprengelmeyer Q. D. Mansourian S. Lange J. D. Matute D. R. Cooper B. S. Jirle E. V. (2020). Recurrent Collection of *Drosophila melanogaster* from Wild African Environments and Genomic Insights into Species History. *Mol. Biol. Evol.* 37 627–638. 10.1093/molbev/msz271 31730190 PMC7038662

[B68] Tauber E. Zordan M. Sandrelli F. Pegoraro M. Osterwalder N. Breda C. (2007). Natural selection favors a newly derived timeless allele in *Drosophila melanogaster*. *Science* 316 1895–1898. 10.1126/science.1138412 17600215

[B69] Umina P. A. Weeks A. R. Kearney M. R. Mckechnie S. W. Hoffmann A. A. (2005). A rapid shift in a classic clinal pattern in *Drosophila* reflecting climate change. *Science* 308 691–693. 10.1126/science.1109523 15860627

[B70] Veeramah K. R. Brud E. Eanes W. F. (2020). Florida *Drosophila melanogaster* genomes sampled 13 years apart show increases in warm-associated SNP alleles. *bioRxiv* [Preprint]. 10.1101/2020.10.23.352732

[B71] Waldvogel A. M. Feldmeyer B. Rolshausen G. Exposito-Alonso M. Rellstab C. Kofler R. (2020). Evolutionary genomics can improve prediction of species’ responses to climate change. *Evol. Lett.* 4 4–18. 10.1002/evl3.154 32055407 PMC7006467

[B72] Wandeler P. Hoeck P. E. Keller L. F. (2007). Back to the future: museum specimens in population genetics. *Trends Ecol. Evol.* 22 634–642. 10.1016/j.tree.2007.08.017 17988758

